# An Investigation of *Enterococcus* Species Isolated from the African Buffalo (*Syncerus caffer*) in Serengeti National Park, Tanzania

**DOI:** 10.1264/jsme2.ME17025

**Published:** 2017-10-28

**Authors:** Eunju Shin, Simon Mduma, Julius Keyyu, Robert Fyumagwa, Yeonhee Lee

**Affiliations:** 1 Culture Collection of Antimicrobial Resistant Microbes, Department of Horticulture, Biotechnology, and Landscape Architecture, Seoul Women’s University Seoul 01797 Korea; 2 Tanzania Wildlife Research Institute P.O. Box 661, Arusha Tanzania

**Keywords:** African buffalo, *Enterococcus* species, antimicrobial susceptibility, MLST, phylogeny

## Abstract

We isolated *Enterococcus* species that colonized in the African buffalo (*Syncerus caffer*) in order to investigate their genetic relatedness and antimicrobial susceptibility. A total of 219 isolates were obtained and a 16S rRNA gene sequence analysis showed they were classified into *Enterococcus avium*, *E. casseliflavus*, *E. faecalis*, *E. faecium*, *E. hirae*, or *E. mundtii*. Multilocus sequence typing of *E. faecalis* and *E. faecium* isolates indicated that some of the isolates showed an evolutionary distance that was far from the primary founders. The antimicrobial susceptibility of the enterococcal isolates suggested that the significant transmission of antimicrobial resistance via human intervention had not yet occurred.

Human and animal microbiomes have developed resistance to commonly used antimicrobials; however, most antimicrobial resistance studies have focused on humans and domestic animals, and not on wild animals (3, 26). Limited information is currently available on the gut microbiome of animals living in a wildlife protection ecosystem.

Serengeti National Park is known for its incredible scenery and magnificent wildlife. It covers 14,763 km^2^ (1°28′−3°17′ S, 33°05′−35°20′ E) in northern Tanzania (altitude of 910 to 1820 m a.s.l). The Serengeti ecosystem is a part of Tanzania’s protected area, which encompasses a geographical region of wildebeest migration (10). The African buffalo (*Syncerus caffer*) is a key species in the Serengeti ecosystem, and these animals roam freely in Serengeti National Park. They graze on various grass species, and their movement within the park depends on water and grass availability (10). They interact with humans and occasionally with livestock via common water sources and grazing areas. Human–wildlife contact has increased concomitantly with increments in human populations and travel (26).

Since enterococci and *Escherichia coli* occur within the gut microbiome of humans and animals (1, 13, 31) and easily acquire antimicrobial resistance, they may serve as an indicator organism for the emergence of antimicrobial resistant bacteria (29). We selected commensal bacteria, particularly enterococci, colonizing the gastrointestinal tract of African buffaloes to survey antimicrobial resistance in wild ecosystems. Enterococci are non-spore-forming Gram-positive facultative anaerobic bacteria with spherical cocci appearing separately, in pairs, or in short chains (27). Most enterococci are not virulent and are considered to be relatively harmless, with little potential for human infection (27). However, they have occasionally been identified as nosocomial opportunistic pathogens that exhibit increased resistance to antimicrobials (22). Antimicrobial resistance in enterococci may be divided into two classes: intrinsic resistance and acquired resistance. Intrinsic resistance is due to either a lack of target sites for antimicrobials or the insufficient penetration of antimicrobials to the intracellular target. Acquired resistance arises from genetic mutations or the addition of extrinsic genes. Intrinsic and acquired resistance both hinder the treatment of *Enterococcus* infection (20, 23). Our study was prompted by concerns of the transfer risk of antimicrobial resistance because of human intervention within the wild animal habitat. This study also aimed to increase our understanding of *Enterococcus* species that colonized in the African buffalo. In order to achieve this, we used molecular techniques and antimicrobial susceptibility testing, with a focus on genetic relatedness among *Enterococcus faecalis* and *E. faecium* based on multilocus sequence typing (MLST).

During routine surveillance for foot and mouth disease and Rift Valley fever virus in July 2012, 105 ear swab samples and 104 rectal swab samples were collected from healthy African buffaloes in the Seronera region in the Serengeti, in which various wild fauna are present. Ear and rectal samples were obtained using cotton swabs, which were placed in Amies transport media without charcoal (Yuhan Lab Tech, Seoul, Korea) after sample collection. Samples were kept in a cold container and delivered to the laboratory in the Tanzania Wildlife Research Institute (TAWIRI) according to the rules of the UN recommendations on the transport of dangerous goods category 2 and then maintained at −20°C until transported. Samples were delivered via air freight in a cold container, which took approximately 48 h. Upon arrival, samples were streaked onto Enterococcosel agar (BBL Becton Dickinson, Sparks, MD, USA). After a 48-h incubation at 37°C, black colonies with a different colony morphology from other colonies on the plate were subcultured on a new Enterococcosel agar plate and trypticase soy agar (BBL) with 5% defibrinated sheep blood and incubated for 24 h at 37°C. After cell morphology was examined under a microscope, each isolated colony was stored at −70°C in 20% glycerol and deposited at the Culture Collection of Antimicrobial Resistant Microbes (CCARM, Seoul, Korea). Bacterial cells grown on tryptic soy agar (BBL) for 24 h at 37°C were suspended in sterile saline and then harvested by centrifugation at 14,000×*g* for 10 min. Genomic DNA was extracted in 30 μL of 10 mM TE buffer (10 mM Tris-Cl, 1 mM EDTA, pH 8.5) using a G-spin genomic extraction kit (Intron Biotechnology, Seoul, Korea) and stored at −20°C until analyzed. PCR of the 16S rRNA gene was performed with 16S rRNA primers (27F, 5′-AGA GTT TGA TCM TGG CTC AG-3′; 1088R, 5′-GCT CGT TGC GGG ACT TAA CC-3′) to amplify the coding region within the 16S rRNA gene (21). DNA fragments were purified using the QIAquick Gel extraction Kit (Qiagen, Valencia, CA, USA) in accordance with the manufacturer’s instructions, and sequence reactions were performed with an ABI 3100 automated sequencer (Applied Biosystems) by Bionix (Seoul, Korea). DNA sequences were analyzed using the online BLAST algorithm at the National Center for Biotechnology Information web server (http://www.ncbi.nlm.nih.gov) and the EzTaxon server (http://www.ezbiocloud.net/identify) (18) based on 16S rRNA gene sequence data.

Minimal inhibitory concentrations (MICs) were assayed on Mueller–Hinton agar (MH, BBL) in accordance with the recommendations of the Clinical Laboratory Standards Institute (6). The following 10 antimicrobials were tested: ampicillin, chloramphenicol, ciprofloxacin, erythromycin, gentamicin, norfloxacin, synercid, teicoplanin, tetracycline, and vancomycin. All chemicals were purchased from Sigma (St. Louis, MO, USA), except for synercid (May & Baker, Guildford, UK) and teicoplanin (Hoechst Marion Roussel, Kansas City, MO, USA). *Enterococcus faecalis* ATCC 29212 was included in each batch of the agar dilution test, and CLSI-approved MIC quality-control limits for *E. faecalis* were used. MIC was assessed following the criteria of *Enterococcus* species by CLSI (7, 8). Vancomycin MIC higher than 8 μg mL^−1^ by the agar dilution method was confirmed by Etest^®^ (BioMérieux SA, Marcy l’Etoile, France).

PCR amplification was performed using primers specific to seven housekeeping genes of *E. faecalis* (*gdh* for glucose-6-phosphate dehydrogenase, *gyd* for glyceraldehyde-3-phosphate dehydrogenase, *pstS* for the phosphate ATP-binding cassette transporter, *gki* for glucokinase, *aroE* for shikimate-5-dehydrogenase, *xpt* for xanthine phosphoribosyltransferase, and *yiqL* for acetyl-CoA acetyltransferase) according to the methods of Ruiz-Garbajosa *et al.* (28). Regarding *E. faecium*, primers specific to seven housekeeping genes (*adk* for adenylate kinase, *atpA* for the ATP synthase, alpha subunit, *ddl* for D-alanine:D-alanine ligase, *gyd* for glyceraldehyde-3-phosphate dehydrogenase, *gdh* for glucose-6-phosphate dehydrogenase, *purK* for the phosphoribosylaminoimidazol carboxylase ATPase subunit, and *pstS* for the phosphate ATP-binding cassette transporter) were used according to the method of Homan *et al.* (16). Amplified products were sequenced in both directions using the PCR primers by Bionix. Sequence types (STs) were identified by comparing the sequences with alleles from the *E. faecalis* and *E. faecium* MLST database (http://www.mlst.net). New alleles and STs were submitted to the database curator for assignment. MLST datasets were analyzed using the eBURST algorithm to assess evolutionary relatedness among ST variants. Groups of STs sharing five out of seven alleles were defined as belonging to the same eBURST group, as described elsewhere (32). All available STs of *E. faecalis* and *E. faecium* in the MLST database were simultaneously analyzed and illustrated as population snapshots using the eBURST algorithm (11).

A total of 219 presumptive *Enterococcus* isolates were obtained from 105 ear swab samples and 104 fecal swab samples from African buffaloes. Ninety-six colonies grew from ear samples and 123 colonies from fecal samples (Table S1 and S2). *Enterococcus* colonies were white or yellow in color on trypticase soy agar with 5% defibrinated sheep blood. Bacterial cells of these colonies were found to be cocci when examined under a microscope after Gram staining. The 16S rRNA gene sequences of the isolates were classified into six groups and the phylogenetic tree indicated that they belonged to *E. avium*, *E. casseliflavus*, *E. faecalis*, *E. faecium*, *E. hirae*, and *E. mundtii* respectively, in the genus *Enterococcus* (Table 1 and Fig. S1)

The MICs of *Enterococcus* species from African buffaloes are shown in Table 1. All enterococci isolates, except for two (*E. casseliflavus* CCARM 5297 and *E. mundtii* CCARM 5284), were 100% susceptible to ampicillin, chloramphenicol, ciprofloxacin, erythromycin, teicoplanin, tetracycline, and vancomycin. The MICs of *E. casseliflavus* CCARM 5297 and *E. mundtii* CCARM 5284 against norfloxacin were 16 μg mL^−1^, which indicates resistance according to the CLSI criteria (≥16 μg mL^−1^).

Thirteen STs were obtained from 19 isolates of *E. faecalis*. Among these, two STs were found in the existing database, while 11 STs were new STs (Table 2). The 11 new STs were assigned in the *E. faecalis* PubMLST database (http://pubmlst.org/efaecalis/). Five out of 11 STs (ST-648, ST-650, ST-653, ST-654, and ST-667) were new combinations of previously registered alleles. Six STs (ST-649, ST-651, ST-652, ST-665, ST-666, and ST-668) were new combinations of previously registered alleles with the new allele sequences. Nine out of 13 new STs were represented by a single isolate. Two existing STs (ST-273 and ST-436) were identical to the STs assigned to fecal isolates from non-hospitalized individuals in Germany and France, respectively (http://pubmlst.org/efaecalis). Sixteen isolates of *E. faecium* yielded four novel STs (Table 2). These STs were submitted to the MLST database (http://pubmlst.org/efaeium/) and designated as ST-1081, ST-1082, ST-1083, and ST-1084. In accordance with the same eBURST group defined as groups of STs sharing five out of seven alleles, 74 eBURST groups and 136 singletons of the entire *E. faecalis* STs were obtained, and the 13 STs from 19 *E. faecalis* isolates belonged to seven eBURST groups and three singletons (Table 2). Sixteen groups and 49 singletons were produced from all *E. faecium* STs. ST-1084 belonged to eBURST group 1, ST-1081 and ST-1082 belonged to eBURST group 8, and ST-1083 was a singleton. *E. faecalis* and *E. faecium* MLST databases are displayed as a single eBURST diagram by setting the group definition to zero of seven shared alleles (Fig. S2 and S3).

Advanced molecular technologies have led to considerable changes in the taxonomy of the genus *Enterococcus*, which currently consists of 35 recognized species, with eight more species to be added (27). Enterococci may reside in the gut of animals, and play an important role in nutrition and health (29). Human and animal gastrointestinal tracts represent reservoirs for enterococci (27). A wide variety of *Enterococcus* spp. have been recovered from humans and various animals, and differ greatly from each other (17). In general, the predominant species in fecal samples of healthy humans and animals are *E. faecium* followed by *E. faecalis*. In human infections, *E. faecalis* was the most frequently detected *Enterococcus* species until the mid-1990s. *E. faecium* has increased, possibly because of the spread of antimicrobial resistance, which particularly affects the incidence of nosocomial infection (22, 25, 31). For example, *E. faecium* has become a *vanA*-type (vancomycin-resistant, teicoplanin-susceptible) strain with the increased use of the antimicrobial vancomycin, and has better survival than *E. faecalis*. Other species, including *E. durans*, *E. gallinarum*, and *E. casseliflavus*, are less common isolates (31). *E. avium*, *E. durans*, *E. gallinarum*, *E. casseliflavus*, *E. hirae*, *E. mundtii*, and *E. raffinosus* have also been isolated from human infections at low frequencies (14, 31). In isolates from plants, *E. casseliflavus*, *E. mundtii*, and *E. sulfureus* (9, 22) with a yellow pigment have commonly been detected (2). Since the African buffalo is a herbivore, the high frequencies of *E. casseliflavus* and *E. mundtii* detected in this study may be related to their diet. In comparison with these species, *E. faecalis* (8.7%) and *E. faecium* (7.3%) showed markedly lower isolation rates from the African buffalo than *E. casseliflavus* and *E. mundtii*, which is inconsistent with previous findings (24, 30, 31). We presumed that the distributions of enterococci vary in different animals according to the species, geographical region, and environmental conditions, such as diet.

In the present study, antimicrobial susceptibility test results revealed no resistant *Enterococcus* isolates, except for two norfloxacin-resistant isolates with MICs of 16 μg mL^−1^. Since fluoroquinolones show poor or moderate activity against enterococci and fluoroquinolones are rarely used in the treatment of *Enterococcus* infection (4, 17), these two isolates do not appear to be important. However, because fluoroquinolone resistance may be transferred to other genera, further studies to characterize the two fluoroquinolone-resistant strains are underway. Fluoroquinolone resistance may occur under the following conditions: a single mutation in the *gyrA* gene; decreased accumulation in bacteria due to mutations in the outer membrane proteins; and efflux pumps and/or plasmid-mediated resistance *qnr* gene (4, 19, 33). Since enterococci are facultative anaerobes, they have intrinsic low-level resistance to aminoglycosides via limited drug uptake, which is associated with the proteins involved in electron transport, with MICs ranging between 4 μg mL^−1^ and 256 μg mL^−1^ (5, 15, 23). Our results show that the antimicrobial susceptibilities of *Enterococcus* isolates from the African buffalo were significantly lower than those reported for others (*e.g.*, humans, livestock, companion animals, and wildlife animals) (2, 10, 12, 20). Acquired antimicrobial resistance, which has been reported in human-derived enterococci, was not found in these isolates.

The MLST database for *E. faecalis* and *E. faecium* as of December 2016 contains 744 STs and 1275 STs from global collections, respectively. Population snapshots of entire STs in the MLST database showed evolutionary distance among ST variants, and *E. faecalis* represented a more complicated snapshot than *E. faecium* (Fig. S2 and S3). A collection of 19 *E. faecalis* isolates yielded 11 novel STs, with the exception of STs. According to the eBURST analysis, ST-436 was clearly derived from the primary founder ST-6, whereas ST-648 may have descended from ST-6. In both species, 15 novel STs were recovered, and they showed an evolutionary distance far from the primary founders, which is defined as the ST that differs from the largest number of other STs at a single locus only (11, 32). Several genetic elements, such as conjugative plasmids and conjugative transposons, are generally known to be involved in genetic transfer.

In conclusion, the distribution of *Enterococcus* species in the African buffalo was not similar to that previously reported in humans, livestock, and wildlife (3, 31). Our results showed that the most frequently isolated *Enterococcus* species in the African buffalo in the Serengeti ecosystem was *E. casseliflavus*, followed by *E. mundtii*, which is known to be prevalent in plants. The antimicrobial susceptibilities and molecular characteristics of the *Enterococcus* isolates in the present study differed greatly from those from different sources and countries (2, 10, 12, 20). These differences may have arisen from the location and environment at which samples were acquired. Our results showed no evidence of antimicrobial resistance transfer in the protected area of the Serengeti ecosystem by human intervention. However, increased human activities within the habitats of wild animals, such as wildlife habitat exploitation, trophy hunting, tourism, livestock keeping, and agriculture, may increase the risk of the transmission of antimicrobial-resistant species and resistance genes. This study is the first to report *Enterococcus* species in the wild African buffalo in Tanzania with the application of an MLST analysis and antimicrobial resistance assays.

## Supplemental data



## Figures and Tables

**Table 1:** Proportion and minimal inhibitory concentrations of *Enterococcus* species isolated from ear and rectal swab samples of the African buffalo (*Syncerus caffer*) in the Serengeti, Tanzania.

Species (no. of isolates)	No. of isolates (%)			MIC (μg mL^−1^) range^a^									
	
	Ear	Fecal	Total	AMP (≥16)	CM (≥32)	CIP (≥4)	EM (≥8)	GM (≥500)	NOR (≥16)	SYN (≥4)	TEI (≥32)	TET (≥16)	VAN (≥32)
*E. avium*	6 (6.3)	0 (0)	6 (2.7)	≤0.25–1	4–8	1–2	≤0.25	0.5–4	1–4	1–4	0.13–2	≤0.25–1	≤0.5–4
*E. casseliflavus*	67 (69.8)	43 (35.0)	110 (50.2)	≤0.25–1	4–8	0.5–2	≤0.25–4	1–8	2–16	0.5–8	0.06–4	≤0.25–1	1–8
*E. faecalis*	12 (12.5)	7 (5.7)	19 (8.7)	≤0.25–2	4–8	0.5–2	≤0.25–1	2–32	1–8	2–16	0.06–4	0.5–1	1–8
*E. faecium*	1 (10)	15 (12.2)	16 (7.3)	≤0.25–4	4–8	0.5–2	≤0.25–4	2–8	1–8	≤0.25–2	0.25–4	≤0.25–0.5	≤0.5–8
*E. hirae*	0 (0)	3 (2.4)	3 (1.4)	1–2	4–8	≤0.25–1	≤0.25	2–8	1–4	1–2	0.13–0.5	≤0.25–1	1
*E. mundtii*	10 (10.4)	55 (44.7)	65 (29.7)	≤0.25–1	4–8	0.5–2	≤0.25–1	1–8	2–16	≤0.25–8	≤0.03–4	≤0.25–1	≤0.5–12
Total	96 (100)	123 (100)	219 (100)	≤0.25–4	4–8	≤0.25–2	≤0.25–4	0.5–32	1–16	≤0.25–8	≤0.03–4	≤0.25–1	≤0.5–12
% Susceptible^b^				100	100	100	100	100	99.1^c^	100	100	100	100

a AMP, ampicillin; CM, chloramphenicol; CIP, ciprofloxacin; EM, erythromycin; GM, gentamicin; NOR, norfloxacin; SYN, synercid; TEI, teicoplanin; TET, tetracycline; VAN, vancomycin. Values in parentheses are the criterion limits for resistance according to the Clinical Laboratory Standards Institute (CLSI).

b Proportion of isolates that are susceptible to an antimicrobial.

c Two isolates *E. casseliflavus* CCARM 5297 and *E. mundtii* CCARM 5284 detected from a fecal sample were 16 μg mL^−1^.

**Table 2:** Multilocus sequence types of 19 *Enterococcus faecalis* and 16 *E. faecium* isolates from the African buffalo (*Syncerus caffer*) in the Serengeti, Tanzania.

*E. faecalis* isolates (*n*=19)									

ST^a^	Allele^b^							No. of isolates	eBURST group^c^

	*gdh*	*gyd*	*pstS*	*gki*	*aroE*	*xpt*	*yiqL*		
ST-273	4	8	12	13	40	46	22	1	26
ST-436	12	7	3	17	6	20	5	1	1
**ST-648**	12	10	3	17	31	2	5	3	1
**ST-649**	15	2	***80***	1	3	15	11	3	4
**ST-650**	17	7	16	5	75	1	9	1	singleton
**ST-651**	***79***	5	***79***	***81***	***85***	***73***	***79***	2	50
**ST-652**	34	1	***81***	***82***	3	***74***	***79***	1	singleton
**ST-653**	4	28	17	37	29	23	17	1	20
**ST-654**	20	1	14	15	3	14	9	2	52
**ST-665**	***80***	6	7	16	11	11	11	1	16
**ST-666**	12	5	***79***	***81***	***85***	***73***	***79***	1	50
**ST-667**	14	8	12	13	31	46	22	1	26
**ST-668**	81	1	17	6	***85***	14	28	1	singleton

*E. faecium* isolates (*n*=16)									

*ST*	Allele							No. of isolates	eBURST group

	*atpA*	*ddl*	*gdh*	*purK*	*gyd*	*pstS*	*adk*		
**ST-1081**	2	9	***72***	7	22	***100***	1	1	8
**ST-1082**	2	9	1	7	22	***100***	1	8	8
**ST-1083**	20	***77***	6	***81***	1	***101***	1	1	singleton
**ST-1084**	62	9	1	7	1	1	1	6	1

a ST, sequence type. New STs identified in this study are in boldface.

b New alleles identified in this study are written in italics and boldface.

c STs sharing five out of seven alleles are defined as the same eBURST group.
