# Supplementary material for: An Investigation of Enterococcus Species Isolated from the African Buffalo (Syncerus caffer) in Serengeti National Park, Tanzania
Source: Microbes Environ. 2017 Oct 28;32(4):402–6. doi: 10.1264/jsme2.ME17025 (PMC5745028; doi:10.1264/jsme2.ME17025)
Supplement: Supplementary file 1 [file 32_402_s1.pdf]

**Supplemental data**

Table S1. Number of samples and isolates

| Source | No. of samples | No. of isolates | positive[a]/negative[b] |
|---|---|---|---|
| rectal | 104 | 123 | 85/19 |
| ear | 105 | 96 | 88/17 |
| total | 209 | 219 | 172/36 |

[a] number of samples which yielded Enterococcus isolate
[b] number of samples which not yielded Enterococcus isolate

Table S2. Number of isolates per sample

| Rectal | | Ear | |
|---|---|---|---|
| Sample No. | No. of isolate | Sample No. | No. of isolate |
| 1 | 2 | 105 | 0 |
| 2 | 2 | 106 | 1 |
| 3 | 0 | 107 | 1 |
| 4 | 1 | 108 | 1 |
| 5 | 1 | 109 | 0 |
| 6 | 1 | 110 | 0 |
| 7 | 2 | 111 | 1 |
| 8 | 2 | 112 | 1 |
| 9 | 2 | 113 | 1 |
| 10 | 1 | 114 | 1 |
| 11 | 2 | 115 | 1 |
| 12 | 2 | 116 | 1 |
| 13 | 1 | 117 | 0 |
| 14 | 1 | 118 | 1 |
| 15 | 2 | 119 | 1 |
| 16 | 2 | 120 | 1 |
| 17 | 2 | 121 | 0 |
| 18 | 3 | 122 | 1 |
| 19 | 1 | 123 | 1 |
| 20 | 1 | 124 | 0 |
| 21 | 0 | 125 | 1 |
| 22 | 2 | 126 | 1 |
| 23 | 1 | 127 | 1 |
| 24 | 2 | 128 | 2 |
| 25 | 2 | 129 | 1 |
| 26 | 0 | 130 | 1 |
| 27 | 1 | 131 | 2 |
| 28 | 1 | 132 | 1 |
| 29 | 1 | 133 | 1 |
| 30 | 1 | 134 | 1 |
| 31 | 1 | 135 | 0 |
| 32 | 1 | 136 | 1 |
| 33 | 2 | 137 | 1 |
| 34 | 2 | 138 | 1 |
| 35 | 1 | 139 | 0 |
| 36 | 1 | 140 | 1 |
| 37 | 1 | 141 | 0 |
| 38 | 0 | 142 | 1 |
| 39 | 2 | 143 | 1 |
| 40 | 1 | 144 | 1 |
| 41 | 2 | 145 | 1 |
| 42 | 1 | 146 | 2 |
| 43 | 0 | 147 | 2 |
| 44 | 1 | 148 | 0 |
| 45 | 1 | 149 | 1 |
| 46 | 2 | 150 | 2 |
| 47 | 2 | 151 | 1 |
| 48 | 2 | 152 | 1 |
| 49 | 2 | 153 | 1 |
| 50 | 0 | 154 | 2 |
| 51 | 2 | 155 | 1 |
| 52 | 1 | 156 | 0 |
| 53 | 2 | 157 | 1 |
| 54 | 1 | 158 | 1 |
| 55 | 1 | 159 | 1 |
| 56 | 1 | 160 | 0 |
| 57 | 0 | 161 | 1 |
| 58 | 1 | 162 | 1 |
| 59 | 1 | 163 | 1 |
| 60 | 2 | 164 | 0 |
| 61 | 1 | 165 | 1 |

| | | | |
|---|---|---|---|
| 62 | 1 | 166 | 1 |
| 63 | 1 | 167 | 1 |
| 64 | 2 | 168 | 1 |
| 65 | 1 | 169 | 1 |
| 66 | 1 | 170 | 1 |
| 67 | 2 | 171 | 1 |
| 68 | 2 | 172 | 1 |
| 69 | 1 | 173 | 1 |
| 70 | 1 | 174 | 1 |
| 71 | 2 | 175 | 1 |
| 72 | 2 | 176 | 1 |
| 73 | 1 | 177 | 1 |
| 74 | 2 | 178 | 1 |
| 75 | 0 | 179 | 1 |
| 76 | 2 | 180 | 0 |
| 77 | 2 | 181 | 1 |
| 78 | 2 | 182 | 1 |
| 79 | 1 | 183 | 1 |
| 80 | 0 | 184 | 1 |
| 81 | 0 | 185 | 1 |
| 82 | 0 | 186 | 1 |
| 83 | 0 | 187 | 1 |
| 84 | 0 | 188 | 1 |
| 85 | 0 | 189 | 1 |
| 86 | 0 | 190 | 1 |
| 87 | 0 | 191 | 1 |
| 88 | 0 | 192 | 1 |
| 89 | 0 | 193 | 1 |
| 90 | 0 | 194 | 2 |
| 91 | 1 | 195 | 1 |
| 92 | 2 | 196 | 2 |
| 93 | 1 | 197 | 0 |
| 94 | 1 | 198 | 1 |
| 95 | 1 | 199 | 1 |
| 96 | 1 | 200 | 1 |
| 97 | 1 | 201 | 0 |
| 98 | 2 | 202 | 2 |
| 99 | 1 | 203 | 0 |
| 100 | 1 | 204 | 1 |
| 101 | 1 | 205 | 1 |
| 102 | 1 | 206 | 0 |
| 103 | 2 | 207 | 1 |
| 104 | 1 | 208 | 1 |
| | | 209 | 1 |

7

**Supplemental data**

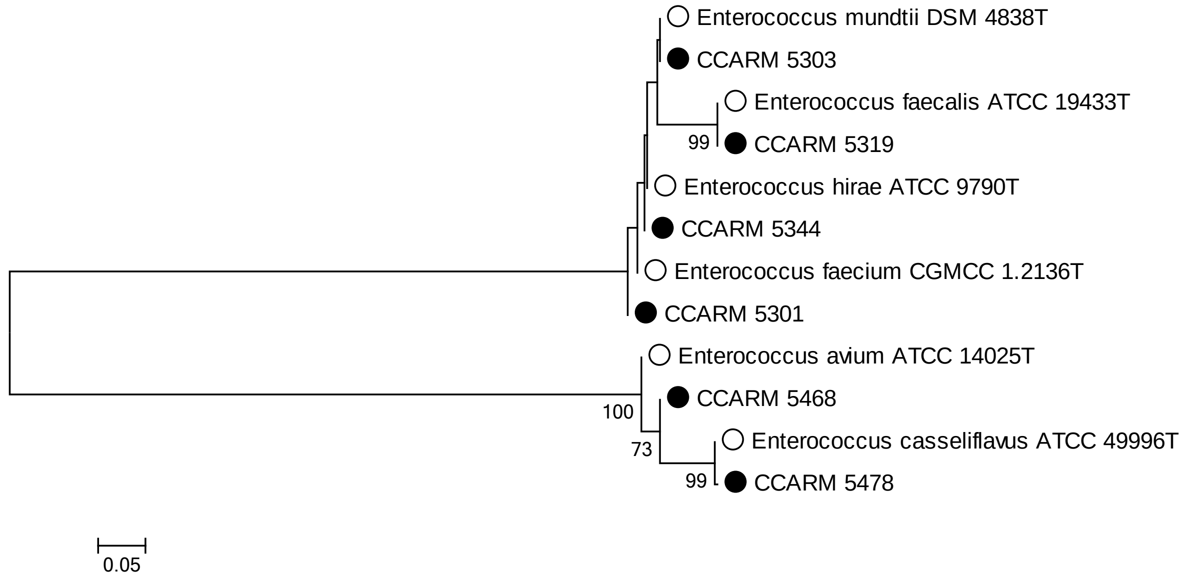


**Fig S1.** A neighbor-joining tree, based on 16S rRNA gene sequences, showing the phylogenetic relatedness of the strains found in this study. The tree was constructed based on partial nucleotide sequences excluding the primer (27f, 1088r)-binding regions using the program MEGA6. A bootstrap analysis was performed with 1,000 trials and value greater than 70% are indicated. Open circles indicate the type strains and closed circles indicate the representative strains of each group found in this study. Bar, 0.05 substitutions per nucleotide position.

.

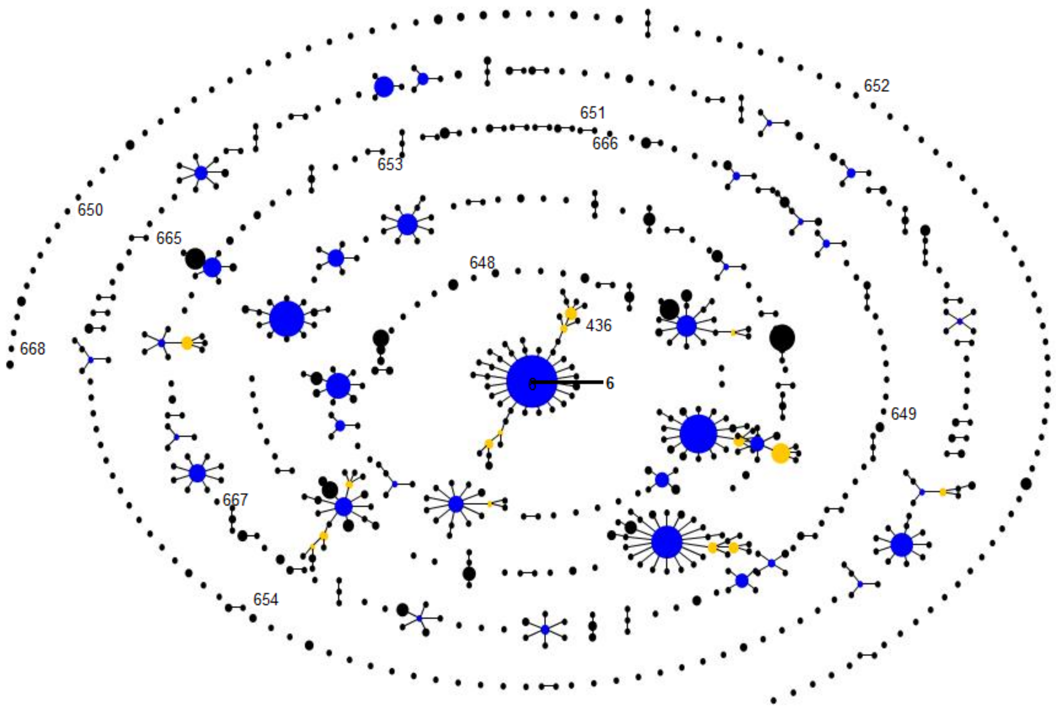


**Fig. S2.** Population snapshot of 19 *Enterococcus faecalis* isolates. Clusters of related sequence types (STs) and individual unlinked STs with the entire *E. faecalis* MLST database are displayed as a single eBURST diagram by setting the group definition to zero of seven shared alleles. Primary founders (blue) are positioned centrally in the cluster, and subgroup founders are shown in yellow. STs found in this study are labeled and the other ST labels have been removed.

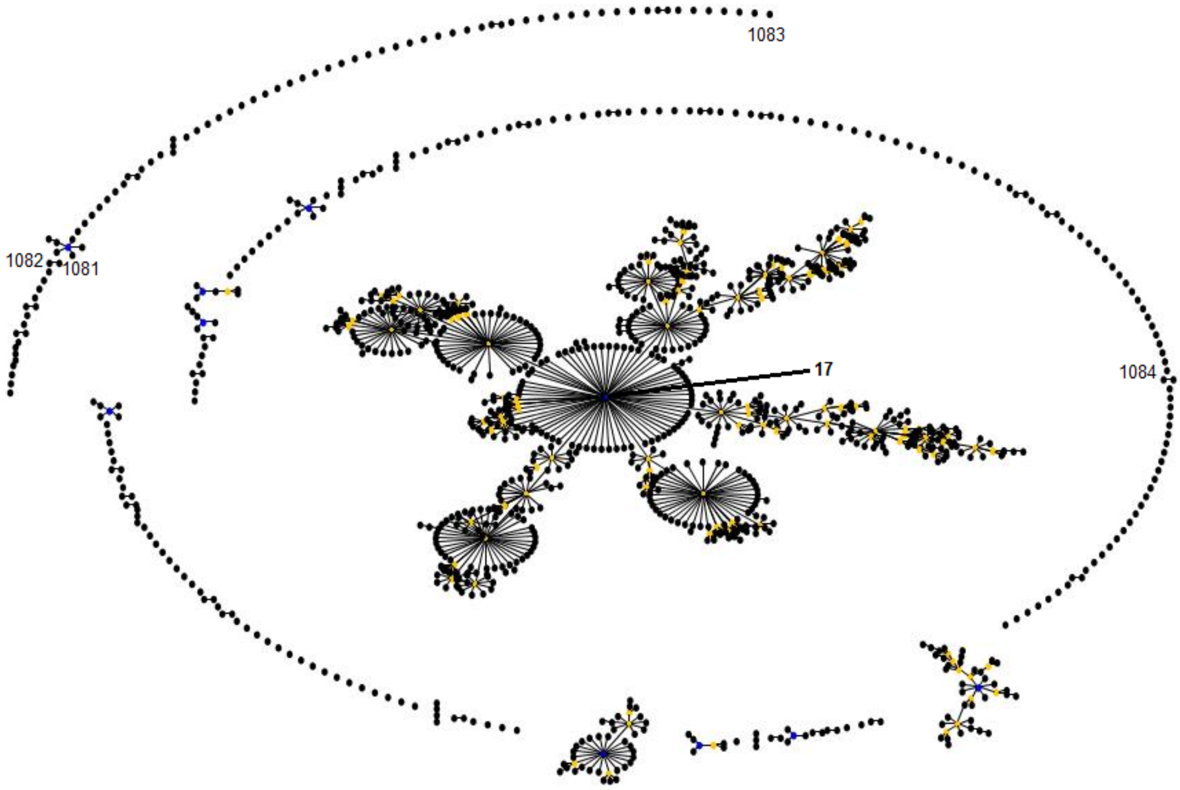


**Fig. S3.** Population snapshot of 16 *Enterococcus faecium* isolates based on eBURST analysis. Clusters of related sequence types (STs) and individual unlinked STs with the entire *E. faecium* MLST database are displayed as a single eBURST diagram by setting the group definition to zero of seven shared alleles. Primary founders (blue) are positioned centrally in the cluster, and subgroup founders are shown in yellow. STs found in this study are labeled and the other ST labels have been removed.
